# *Cis*- and *Trans*-Acting Expression Quantitative Trait Loci of Long Non-Coding RNA in 2,549 Cancers With Potential Clinical and Therapeutic Implications

**DOI:** 10.3389/fonc.2020.602104

**Published:** 2020-10-19

**Authors:** Wenzhi Li, Chaoqun Xu, Jintao Guo, Ke Liu, Yudi Hu, Dan Wu, Hongkun Fang, Yun Zou, Ziwei Wei, Zhong Wang, Ying Zhou, Qiyuan Li

**Affiliations:** ^1^Department of Urology, Shanghai Ninth People's Hospital, School of Medicine, Shanghai Jiao Tong University, Shanghai, China; ^2^School of Medicine, National Institute for Data Science in Health and Medicine, Xiamen University, Xiamen, China; ^3^Department of Oncology, Xiamen the Fifth Hospital, Xiamen, China

**Keywords:** long non-coding RNA, tumor immune microenvironment, cancer, instrumental variable analysis, tumor infiltrating lymphocytes, expression quantitative trait loci

## Abstract

Many cancer risk loci act as expression quantitative trait loci (eQTLs) of transcripts including non-coding RNA. Long non-coding RNAs (lncRNAs) are implicated in various human cancers. However, the pathological and clinical impacts of the genetic determinants of lncRNAs in cancers remain largely unknown. In this study, we performed eQTL mapping of lncRNA expression (elncRNA) in 11 TCGA cancer types and characterized the biological processes of elncRNAs in the setting of genomic location, cancer treatment responses, and immune microenvironment. As a result, 10.86% of the *cis*-eQTLs and 1.67% of the *trans*-eQTLs of lncRNA were related to known genome-wide association studies (GWAS) cancer risk loci. The elncRNAs are significantly enriched for those which are previously annotated as predictive of drug sensitivities in cancer cell lines. We further revealed the downstream transcriptomic effectors of eQTL-elncRNA pairs. Our data specifically suggested that the genes affected by eQTL-elncRNA associations are enriched in the immune system processes and eQTL-elncRNA associations influence the constitution of tumor infiltrating lymphocytes. In ovarian cancer, the “rs34631313-AC092580.4” pair was associated with increased fraction of CD8+ T cells and M1 Macrophage; whereas in KIRC, the “rs9546285-LINC00426” pair was associated with increased fraction of CD8+ T cells and a decreased fraction of M2 macrophages. Our findings provide a systematic view of the transcriptomic impacts of the eQTL landscape of lncRNA in human cancers and suggest its strong potential relevance to cancer immunity and treatment.

## Introduction

Transcript abundance is an inheritable quantitative trait which serves as a major intermediate variable to explain the functional background of intergenic trait associated loci (TAL) (1). The germline variants which are associated with the transcript levels are known as “expression quantitative trait loci” (eQTL) (2). The eQTL may act either in*-cis* or in*-trans*, depending on the relative positioning between the loci and the target transcripts (1). The biological mechanisms underlying the functions of *cis*- and *trans*-acting eQTLs for mRNA have been addressed by a plethora of studies in various cell lines and tissues (3, 4). Mapping of eQTLs in tumor tissues provides fundamental clues the functional implications of non-coding, risk-associated variants (5–8). Quite a few cancer risk loci have been proven to be genetic determinants of gene expression. For example, the breast cancer risk loci 1p13.2 (rs11552449) acts in*-cis* to influence the activity of *DCLRE1B*, an evolutionarily conserved gene involved in DNA stability and the repair mechanism of inter-strand cross-link (9–11). Recent studies have reported associations between cancer risk loci and lncRNA expression and empirically verified the functional phenotypical effects with clinical significance (12).

Non-coding RNA (ncRNA) comprises diverse RNA transcripts including microRNA (miRNA), long non-coding RNA (lncRNA), circular RNA (circRNA), and piwi-interacting RNA (piRNA), many of which are known to function as regulators of transcription (13). The altered abundance of ncRNAs affects the cancer transcriptome and the consequent processes of carcinogenesis and tumor development (14). Among the ncRNA species, lncRNAs participate in diverse regulatory activities in the cell, including chromatin structuring and reprogramming *cis*-regulation of enhancers, competing endogenous RNA (ceRNA) networks, immune response, and post-transcriptional regulation of mRNA processing (15–19). The regulatory activities of lncRNAs play a critical role in the immune-related disease and the biological processes which determine the pathological and clinical phenotypes of cancers (20, 21). Growing evidence indicated that dysregulation of lncRNAs involved in the regulation of immune system (22). Yongsheng Li *et al*. demonstrated the immune-associated lncRNAs (ImmLnc) show expression perturbation in cancers and are significantly correlated with immune cell infiltration (23), which suggested that lncRNA play an active role in cancer immunity.

Studying the genetic determinants of lncRNAs is crucial for understanding the etiology of carcinogenesis. For example, recent evidence has suggested that risk-associated loci in prostate cancer act in-*cis* on specific lncRNAs, thereby predisposing cells towards malignancy (24). However, eQTL mapping in cancer is more challenging than that in established cell lines due to tissue heterogeneity. In addition, other somatic changes in the genome, such as somatic copy number alterations and CpG methylation might confound the effect of germline determinants. To control for the confounders in the expression data, several studies used multivariate methods to adjust the eQTL mapping results for the effects of heterogeneity, sample purity, and somatic alterations on transcription activity (25). These methods have yielded valuable associations between germline variants and gene expression in cancer. Recent studies have reported eQTLs for various non-coding transcripts (lncRNA and miRNA) in human cancers (11, 26). Moreover, study of Xia *et al*. demonstrated that lncRNAs are active participants in immune regulation in 33 cancer types (23). However, the extensive biological impacts of cancer eQTLs of lncRNA on the whole cancer transcriptome and the tumor microenvironment and the consequential therapeutic implications have not been thoroughly investigated. In addition, differences in the landscape of genetic determinants of lncRNAs and mRNAs in human cancers have not yet been fully addressed. Hence, we investigated the eQTL landscape of lncRNAs in eleven cancer populations from TCGA and revealed the potential pathological and clinical significance of elncRNAs in cancer immunity and treatment responses using Instrumental Variable Analysis (IV) or called Mendelian Randomization (MR) (27).

Our findings suggest that the downstream targets of eQTL-elncRNA pairs are enriched for immune system pathways and are consistently associated with varied fractions of immune cell types and patient clinical outcomes. In addition, we show that the elncRNAs are significantly enriched for known predictors of the treatment responses. Altogether, our data confirm that elncRNAs are active intermediates between genetic variants and the transcriptional activities in cancers, which further influence the tumor immune microenvironment, treatment responses and clinical outcomes.

## Materials and Methods

### Genotype Data Collection, Imputation, and Processing

To identify the eQTLs across eleven cancer types, we obtained genotype data from the TCGA portal (https://portal.gdc.cancer.gov/), which contains Affymetrix genome-wide human SNP 6.0 array-based genotype data for 898,620 single nucleotide polymorphisms (SNPs). To increase the power for eQTL discovery, we imputed the variants on autosomal chromosomes for all samples in each cancer type using IMPUTE2 algorithm with 1000 Genomes Phase 3 (28) as the reference panel. Then, a two-step procedure of IMPUTE2 were performed, which consists of pre-phasing and imputation of the phased data. After that, the following criteria were used to select SNPs: (i) imputation confidence score, INFO ≥0.5, (ii) minor allele frequency (MAF) ≥5%, (iii) SNP missing rate <5% for best-guess genotypes at posterior probability ≥0.7, (iv) Hardy–Weinberg Equilibrium P-value >1×10^−6^ estimated using the Hardy–Weinberg R package (29).

### Transcript Expression Data Collection and Processing

The lncRNA expression profiles of eleven TCGA cancer types were directly obtained from ‘The Atlas of Noncoding RNAs in Cancer’ (TANRIC) database (2018/06/11 – Version 1.3) (30). The expression levels of 12,727 lncRNAs were quantified as reads per kilobase per million mapped reads (RPKM). To ensure the detection power and reduce the noise, we applied a three-step filtering according to a previous study (31): (1) The lncRNAs with the 10^th^-percentile RPKM value is equal to 0 were excluded, and those with the 90^th^-percentile RPKM value is greater than 0.1 were retained for further analysis. (2) Quantile-normalization of the lncRNA expression matrix ensured that the underlying gene expression distribution was similar for all samples. (3) Finally, we applied an inverse normal transformation to the gene expression matrix, such that the expression levels of each lncRNA across samples was matched to the quantiles of standard normal distribution. The expression value of each lncRNA was also transformed based on log_2_(RPKM + 1).

### Correction for Technical Confounders

The tumor microenvironment is the non-cancerous cells present in and around a tumor. These non-cancerous components of the tumor may play an important role in cancer biology. Therefore, we removed samples whose tumor purity less than 0.6 based on the criteria of prior study (32). In order to account for the possible latent confounding effects on transcript levels, we used the probabilistic estimation of expression residuals (PEER) method to estimate a set of latent covariates for the gene expression levels in each cancer type (25).

### Covariates of Expression

Previous studies have shown that various factors affecting global gene expression confound eQTL-mapping (33). To exclude the effect of population structure on gene expression, we performed principal component analysis (PCA) for each cancer type and selected the CEU (Northern Europeans from Utah) population for further analysis. To regulate the other potential confounders, age, gender, gene-level somatic copy number alterations (SCNA), and CpG methylation levels were included as additional covariates. The copy number changes of a given lncRNA were determined by the segmented copy number scores of the tumor sample and paired-normal tissues obtained from the UCSC Xena database (https://xena.ucsc.edu/). Then, the CpG methylation status of the promoter area of each lncRNA (TSS 200/ TSS 1500) was determined by the respective discretized values obtained using Agilent HumanMethylation450K, with cutoff values 0.2 and 0.6.

### Identification of eQTLs

For each cancer type, the genotype data, expression data, and covariates were processed to three N (genotype, expression, or covariates) × S (samples) matrices with matched sample identifiers. The lncRNA annotation file (hg19) was obtained from the GENCODE database (https://www.gencodegenes.org/). The SNP location (hg19) was downloaded from dbSNP148 (https://www.ncbi.nlm.nih.gov/projects/SNP/) (v148). eQTL analysis was performed according to the Genotype-Tissue Expression (GTEx) eQTL workflow (34) using the R package of “MatrixEQTL” in the linear regression model (35). The *cis/trans*-eQTL analysis was based on MatrixEQTL with PEER factors and other covariates:

(1) $$lncRNA\;expression_{i}=\beta_{0}+\beta_{1}\times G_{i}+\beta_{2}\times Ai+\beta_{3}\times S_{i}+PEER\;factors+\epsilon_{i}$$

where $\epsilon_{i}∼N(0,\sigma^{2})$ is a Gaussian error term; *G_i_* is the genotype of the *i^th^* sample; *A_i_* is the age of the *i^th^* sample; *S_i_* is the sex of the *i^th^* sample. In the *cis*-eQTL analysis, we evaluated the associations between lncRNA expression levels and the genotype at a given SNP locus located within 1 Mb upstream and 1 Mb downstream of lncRNA. *trans*-eQTLs are defined as associations beyond the 2-Mbp interval. The PEER factors were computed using the R package PEER to exclude the effects of confounding variables (such as the “batch” effects) from the gene expression matrix.

To reveal the complete landscape of eQTLs in cancer, we also performed *trans*-analysis for the lncRNAs. A false discovery rate (FDR) cutoff of 0.1 was applied for the significant *cis*-eQTL-elncRNA pairs, and, due to the vast number of tests performed in the *trans*-analysis, we set a threshold for the test P-values as 1 × 10^−7^ for *trans*-eSNP-elncRNA pairs. In addition, Plink’s clump function was used to shorten the list of *cis/trans*-eQTLs. Then, only the most significant *cis/trans*-eSNPs per haplotype block (r^2^=0.2, clump distance =500 kb) was selected to perform the second round of validation based on multivariate regression, wherein DNA methylation and SCNA were adjusted.

(2) $$lncRNA\;expression_{i}=\beta_{0}+\beta_{1}\times cis‐G_{i}+\beta_{2}\times trans‐G_{i}+\beta_{3}\times Ai+\beta_{4}\times S_{i}+\;PEER\;factors+\beta_{5}\times SCNA_{i}+\beta_{6}\times M_{i}+\epsilon_{i}$$

where $\epsilon_{i}∼N(0,\sigma^{2})$ is a Gaussian error term; $cis‐G_{i}$ is the genotype of the most significant *cis*-eQTL per LD block of the $i^{th}$ sample; $trans‐G_{i}$ is the genotype of the most significant *trans*-eQTL per LD block of the $i^{th}$ sample; $A_{i}$ is the age of the $i^{th}$ sample; $S_{i}$ is the sex of the $i^{th}$ sample; $SCNA_{i}$ is the somatic copy number alteration of the $i^{th}$ sample; $M_{i}$ is the median of TSS200/1500 DNA methylation status of the nearest gene in the $i^{th}$ sample. A complete eQTL analysis results are available at https://xcqxcq.github.io/lncRNA/LncRNA.html.

### GWAS-Related eQTLs

Risk SNPs identified in GWAS studies were downloaded from the GWAS catalog (http://www.ebi.ac.uk/gwas/) (36). The GWAS linkage disequilibrium (LD) blocks were extracted from the rAggr database (raggr.usc.edu) based on the following parameters (SNP dataset: 1000 Genomes; r^2^ threshold =0.5; population panel: CEU (Utah Residents with Northern and Western European Ancestry; distance limit: 500 kb). eQTLs that overlap with GWAS cancer-related tagSNPs and LD SNPs (r^2^≥0.5) were identified as GWAS cancer-related eQTLs.

### Functional Annotation of eQTLs

The eQTLs implicated in the analysis were functionally annotated by FUMA, a web-based bioinformatics tool that uses a combination of positional, eQTL and chromatin interaction mapping to prioritize likely causal variants and genes. The functional annotations included ANNOVAR categories and RegulomeDB scores. Moreover, the ANNOVAR categories identified the genomic positions of the SNPs (for example, intron, exon, intergenic) and the associated function. The RegulomeDB is a categorical score based on the information from normal tissue eQTLs and chromatin markers; 1a–7 with low scores indicate a decreased regulatory function. The scores are stated in (**Supplementary Table S1**).

### Meta-Analysis of Epigenetic Markers

We used chromatin-immunoprecipitation sequencing (ChIP-seq) peak data from the ENCODE database. For *cis*-eQTL overlapped with epigenetics modifications, Bedtools was applied (https://bedtools.readthedocs.io/en/latest/content/tools/fisher.html) to calculate the enrichment of the *cis-*eQTLs for epigenetic markers. Then, meta-analysis was performed using the R package (meta) to integrate the fold-enrichment of each epigenetic marker.

### Instrumental Variable Analysis

Instrumental variable (IV) analysis was employed to identify the *cis-*regulated lncRNAs that affect the transcription of protein-coding genes in-*cis*/*trans* according to the study performed by McDowell et al. (37). The genotype of an SNP as the genetic instrument across n individuals (eQTLs) directly affects the independent variable elncRNA in*-cis*, which in turn, affects the dependent variable mRNA in-*cis*/*trans*. Thus, the IV analysis was performed using the most significant eQTL-lncRNA pairs from the *cis*-analyses on the “AER” package in R-3.5.0, wherein the eQTLs are the genetic instrument, *cis*-lncRNA expression is the “mediator,” and the *cis/trans*-mRNA expression is the “outcome” (38). We obtained the mRNA expression data of the corresponding TCGA samples from UCSC Xena (https://xena.ucsc.edu). To reduce the burden of multiple statistical tests, we filtered the mRNA in each cancer type by the following criteria: (1) Coefficient of variation (CV) >0.5, the 30^th^ percentile log_2_ (count + 1) value >0 and the 90^th^ percentile log_2_ (count + 1) >5. (2). The absolute value of Spearman’s correlation between lncRNA and mRNA >0.3.

IV analysis of tumor infiltrating immune cells was conducted for the significant eQTL-elncRNA associations identified above. The immune cell fraction measures in TCGA cancer samples are based on published data from CIBERSORT (39). We picked the significant eQTL-elncRNA pairs (FDR<0.1, adjusted R^2^>0.1). Again, eQTL is considered as a genetic instrument and elncRNA as an independent variable. Since the dependent variable immune cell fraction is not normally distributed, it was log_2_ (immune cell fraction + 1) transformed.

### Enrichment Test of elncRNAs for lncRNA Predictors of Anticancer Drug Sensitivity

We obtained drug sensitivity data for lncRNAs from Nath et al.’s (21) study from https://osf.io/m2qja/. We analyzed the following datasets: Effects_CTRP.csv, Pval_CTRP.csv, Effects_GDSC.csv, and Pval_GDSC.csv. We performed Fisher-exact test to assess the enrichment of *cis*-elncRNAs/*trans*-elncRNAs for annotated lncRNA predictors of anti-cancer drug sensitivity. The magnitude of enrichment using odds ratios.

## Results

### eQTL Mapping for 11 TCGA Cancer Types

**Figure 1:**
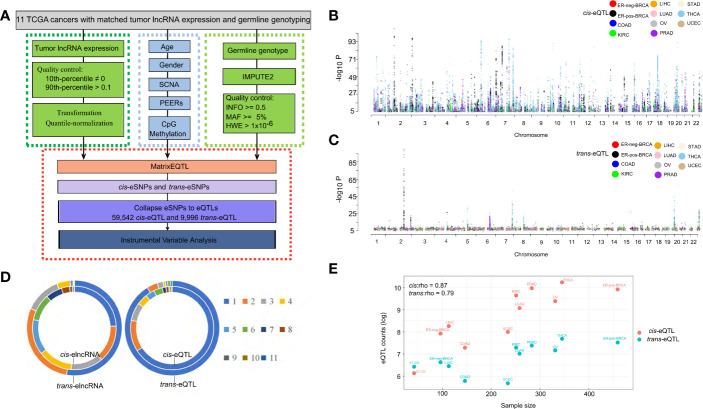
Expression of QTLs in TCGA cancer types. **(A)** Schematic plot showing the eQTL mapping workflow to identify the associations between germline genotype and tumor lncRNA expression in 11 cancer types. **(B)** Manhattan plot of *cis*-eQTLs in 11 cancer types showing -log10 P-values of *cis*-eQTLs in autosomes. Each dot represents a significant eQTL. **(C)** Manhattan plot of *trans*-eQTLs in 11 cancer types showing -log10 P-values of *trans*-eQTLs across the autosome. Each dot represents a significant eQTL. **(D)** Ring diagram showing the common *cis*/*trans*-eQTLs and *cis*/*trans*-elncRNAs across cancer types. “1” represents eQTLs occurring in one cancer type while “11” represents eQTLs occurring in 11 cancer types. **(E)** Log counts of *cis/trans*-eQTLs in each cancer type are positively correlated with sample size.

We performed pan-cancer eQTL mapping for lncRNAs (30) in eleven TCGA cancer types, including ER-pos-BRCA (N=459), ER-neg-BRCA (N=96), COAD (N=146), LUAD (N=249), THCA (N=345), UCEC (N=234), KIRC (N=251), PRAD (N=283), OV (N=331), STAD (N=42), and LIHC (N=113) (**Supplementary Table S2**). The *cis/trans*-eQTL analyses were performed using a two-step regression model. In the first step, we used the MatrixEQTL software (35) to screen for significant expression SNPs (eSNP) (FDR<0.1). Next, to account for linkage disequilibrium (LD), we collapsed the significant eSNPs by the linkage disequilibrium into independent eQTL (r^2^=0.2, clump distance =500 kb). Each eQTL was represented by a tag-eSNP with maximal significance of association. Finally, we verified the significant *cis/trans*-eQTLs using multivariate model adjusting for somatic copy number alteration (SCNA) and DNA methylation in poise *cis*-regulatory regions (TSS-200/TSS-1500, **Figure 1A**).

### eQTL of lncRNAs Expression in Human Cancers

In the *cis*-eQTL analysis, we identified 74,804 significant *cis*-eQTL-lncRNA association pairs from 11 cancer types (FDR<0.1, data is available at https://xcqxcq.github.io/lncRNA/LncRNA.html), which were mapped to 59,542 unique *cis*-eQTLs and 4,742 unique elncRNAs (**Figure 1B** and **Table 1**). In the case of each cancer type, the number of significant *cis*-acting eQTLs varied from 461 (STAD) to 28,085 (THCA) (**Table 1**). The number of the lncRNAs in association with *cis*-eQTL (*cis*-elncRNA) ranged from 98 (STAD) to 2,970 (THCA). As eQTLs are widely reported for mRNA in cancers, we compared the *cis*-eQTLs of lncRNA to those of the mRNA reported previously (40). As a result, the eQTLs of lncRNA and mRNA differ substantially in cancers, with less than 1% (0.01–0.72%) of the mRNA eQTLs are associated with lncRNAs (**Supplementary Table S3** and **Supplementary Figure S1**). Among the 11 cancer types investigated, 226 (KIRC) to 10,087 (THCA) mRNAs were influenced by eQTL as compared to 98 (2.69%, STAD) to 2,970 (83.33%, THCA) lncRNA. For each transcript, the mRNA was affected by 1 to 2 eQTLs, whereas each lncRNA was affected by 5 to 10 eQTLs (**Supplementary Table S3**).

**Table 1:** Cancer type and summary statistics of the eQTL analyses.

Cancer type	Sample size	lncRNA	*cis*- pair	*cis*-eQTL	*cis-* elncRNA	*trans*- pair	*trans*-eQTL	*trans-* elncRNA
ER-neg-BRCA	96	3,434	2,810	2765	553	789	754	490
ER-pos-BRCA	459	3,530	24,866	20,379	2,713	6,408	1,844	677
COAD	146	974	1,487	1,458	313	301	325	190
KIRC	251	3,940	18,055	15,486	2,613	4,918	1,465	725
LIHC	113	2,522	39,53	3,877	817	1,088	634	423
LUAD	249	3,465	9,453	8,761	1,672	1,806	1,114	605
OV	331	3,698	13,575	11,976	2,125	2,965	1,292	607
PRAD	283	3,600	25,906	21,547	2,779	6,459	1,606	671
STAD	42	3,638	464	461	98	496	617	451
THCA	345	3,564	35,637	28,085	2,970	8,380	2,183	666
UCEC	234	1,113	3,074	2,976	527	1,001	292	203
total unique	2549		74,804	59,542	4,742	10,721	9,996	3,284

A majority of the *cis*-eQTLs (65.86%) were cancer type specific. However, a set of *cis*-eQTLs were noted in multiple cancer types. For example, 2.4% *cis*-eQTLs were present in more than eight cancer types, which are termed as “pan-cancer” *cis*-eQTLs hereafter (**Figure 1D**). As for the elncRNAs in-*cis*, 1,150 (24.25%) were cancer-specific, and 237 (5.0%) occurred in more than eight cancer types (**Figure 1D**).

In the *trans*-eQTL analysis, we report 10,721 significant *trans*-eQTL-elncRNA association pairs with genome-wide P-values < 1 × 10^−7^; these pairs were mapped to 9,996 unique *trans*-acting eQTLs and 3,284 unique elncRNAs (**Figure 1C** and **Table 1**). Among the *trans*-eQTLs, 91.26% were cancer-specific, only 0.5% of the *trans-*eQTLs were observed in seven cancer types, suggesting that the *trans*-eQTLs of lncRNA are more cancer-specific (**Figure 1D**). As for the elncRNAs in-*trans*, 1,730 (52.67%) of elncRNAs in-*trans* were cancer-specific (**Figure 1D**), and only 1 (0.03%) elncRNA in*-trans* (RP11-667M19.2) were observed in eight cancer types. Together, these results suggested that that the overall eQTL landscape of elncRNAs are highly specific to the cancer types. However, *trans*-eQTL and their elncRNAs were more cancer-specific than their counterparts in-*cis*.

The eQTL-elncRNA association in cancers features a many-to-many relationship, where one lncRNA can associate with different eQTLs or one eQTL can associate with different elncRNAs. For example, a total of 3,107 elncRNAs were associated with both *cis*- and *trans*-eQTLs in the same cancer type (**Supplementary Table S4**). Moreover, a total of 1,039 eQTLs act both in*-cis* and in-*trans* on different lncRNAs in the same type of cancer (**Supplementary Table S5**). These results suggested that a complex network is involved in the genetic determinants of lncRNA transcription.

Consistent with previous eQTL studies for mRNA (40), the number of significant *cis*-eQTLs for lncRNA identified in the current study was strongly associated with the size of the cohort **(**Spearman’s correlation coefficient, rho=0.87, P=0.00095, **Figure 1E**). A similar tendency was observed in *trans*-eQTLs for lncRNA **(**rho=0.79, P=0.0061, Spearman’s correlation, **Figure 1E**).

### Effect Size of the Determinants of lncRNA Transcript Levels

**Figure 2:**
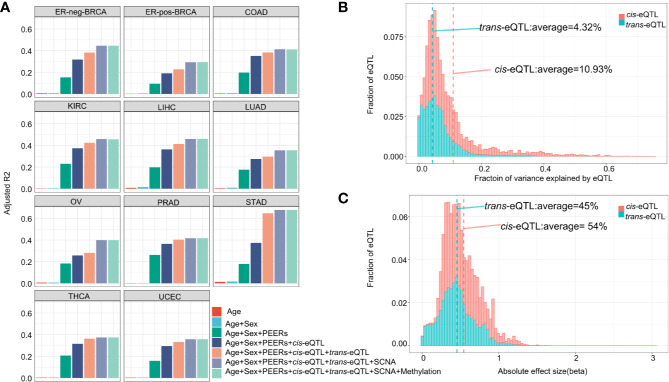
Effect size of eQTLs and other determinants of transcription. **(A)** Cumulative fraction of variance of elncRNA expression was calculated based on sequential addition of the following factors: age, sex, 5 PEER factors, *cis*-eQTL, *trans*-eQTL, somatic copy number alteration and DNA CpG methylation. The bars represent the average fraction of variance for all elncRNAs across 11 TCGA cancer types. Definitions of cancer types are provided in **Table 1**. **(B)** Histograms showing the distribution of the fraction of variance in gene expression explained by the *cis*/*trans*-eQTLs for elncRNAs aggregated across all cancer types. **(C)** Histogram showing the distribution of the absolute effect size (β) of *cis*/*trans*-eQTLs for all cancer types.

The fractions of variation attributed to the major factors of lncRNA expression, including age, sex, PEER factors, gene-level somatic copy number, DNA methylation and *cis*-/*trans*-eQTLs are calculated (**Figure 2A**). A total of five PEER factors were selected to remove the latent confounding effects and maximize the *cis*-elncRNA discovery (**Supplementary Figure S2**). In the 11 cancer types analyzed, *cis*-eQTLs accounted for 7.52% (OV) to 20.15% (STAD) of the total variance in the lncRNA transcript levels, whereas *trans*-eQTLs accounted for 2.16% (LUAD) to 27.76% (STAD) (**Supplementary Figure S3**). The other factors, such as SCNA, explain 1.09% to 11.82% of the total variance, which is much larger than the effect size of CpG methylation (0.01–0.42%) (**Supplementary Figure S3**). However, 9.05% to 26.20% of the variation in lncRNA expression was attributed to non-specific, random effects represented by the PEER factors (**Supplementary Figure S3**). Finally, the effects of age and sex were much smaller than the other factors (**Figure 2A**). Overall, the fractions of variation in lncRNA expression explained by the major factors in cancers are highly similar to that of mRNA, as shown by prior studies (40).

As for the transcript-wise effect sizes, on average, 10.93% of the variance of the elncRNAs expression was attributed to *cis*-eQTLs, compared to 4.32% attributed to *trans*-eQTLs (**Figure 2B**). Notably, the average effect size of *cis*-eQTLs of lncRNA is twice as much as that of the mRNA (5.3%, based on prior eQTL data) (40). For the majority of the elncRNAs, 85.50% *cis*- and 98.16% *trans*-eQTLs accounted for less than 20% of the total variance. However, for a small fraction of the elncRNAs, the effect of genetic determinants mounts to 69.72% (*cis*) and 75.34% (*trans*) (**Figure 2B**). Furthermore, the average allelic effect size of *cis*-eQTLs of lncRNA (fold-change of elncRNA with every unit increase of the effect allele) was 0.54 (**Figure 2C**), which is larger than that of mRNA based on prior study **(**0.37, **Supplementary Table S6**) (40). For all elncRNA in cancers, the allelic effect sizes of the *cis*-eQTLs ranged from 0.002 to 2.71, compared to 3.0×10^−6^ to 3.04 for the *trans*-eQTLs (**Figure 2C**). Taken together, these results suggest that the effect size of genetic determinants on the expression of lncRNA is larger than that of mRNA.

### Functional Characteristics of eQTLs of lncRNAs in Cancers

The majority of genetic determinants of transcripts are localized in intergenic or intronic regions. Thus, we sought to reveal functional characteristics of the eQTLs of lncRNA from three different aspects: the genomic location, the epigenetic landscape and the association with cancer risk-associated loci.

**Figure 3:**
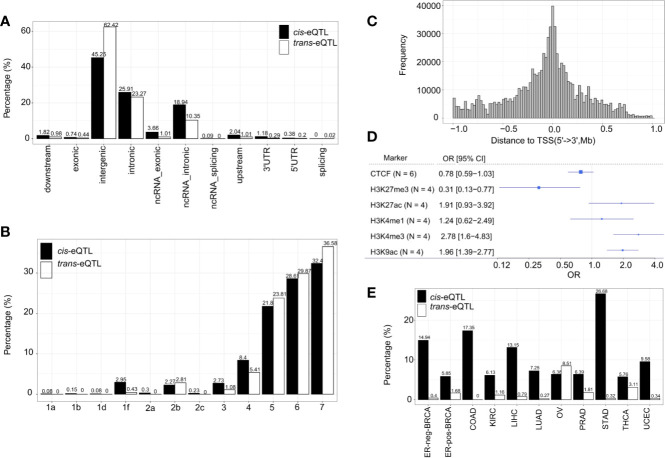
Characterization of *cis*/*trans*-eQTLs of lncRNA. **(A)** Genomic locations of *cis*/*trans*-eQTLs (r^2^≥ 0.2). **(B)** Distribution of RegulomeDB categories of *cis*/*trans*-eQTLs (r^2^≥ 0.2). **(C)** The location distribution of significant *cis*-eQTLs relative to their elncRNAs aggregated across all cancer types. **(D)** Meta-analysis of corresponding cancer cell lines showed significant enrichment of *cis*-eQTLs in H3K27Me3, H3K4Me3, and H3K9Ac. **(E)** The percentage of *cis*/*trans*-eQTLs that are associated with GWAS risk loci in 11 TCGA cancer types.

The genomic locations of the *cis*-eQTLs and *trans*-eQTLs were annotated by Functional Mapping and Annotation of Genome-Wide Association Studies (FUMA) (41). The results demonstrated that the majority of *cis*-eQTLs of lncRNA are located in the intergenic regions (45.25%) and intronic regions (25.91%) (**Figure 3A**). Only a small fraction of *cis*-eQTLs was localized in the exonic (0.74%) or ncRNA exonic (3.66%) regions. The genomic distribution of *cis*-eQTLs of mRNA was similar to that of the *cis*-eQTLs of lncRNA (**Supplementary Figure S4A**). On the other hand, 62.42% of the *trans*-eQTLs of lncRNA were localized in the intergenic regions and 23.27% in the intronic regions (**Figure 3A**); while some were located in the exonic (0.44%) or ncRNA exonic (1.01%) regions. Overall, the genomic distribution of the eQTLs is highly comparable between lncRNA and mRNA.

A number of non-coding QTLs interfered with the regulatory elements of the genome (42). The present study showed that 38.99% of the non-coding *cis*-eQTLs of lncRNAs were co-localized in the transcription factor (TF) binding sites and/or DNase I hypersensitive sites (RegulomeDB score <6) **Figures 3B, C** and **Supplementary Table S1**. Whereas 48.63% of the non-coding *cis*-eQTLs of mRNA were co-localized in the TF binding sites and/or DNase I hypersensitive sites, and 4.95% had a putative regulatory function (**Supplementary Figure S4B**).

Since chromatin structures are tightly associated with the *cis*-regulatory activities, we further evaluated the histone modification landscape of the eQTLs of lncRNA in matched cancer cell lines (**Figure 3D**) and normal tissues (**Supplementary Figure S5A**). As a result, the eQTLs for lncRNA in the matched cancer cell lines were significantly enriched in the active promoter or enhancer markers, such as, H3K4me3 (OR=2.78, 95% CI: 1.60 to 4.83) and H3K27ac (OR=1.91, 95% CI: 0.93–3.92). The eQTLs were also negatively enriched in transcription repressor markers, such as H3K27me3 (OR=0.31, 95% CI: 0.13–0.77) (**Figure 3D**). In the matched normal tissues, the eQTLs of lncRNA showed a consistent tendency of enrichment for the histone markers (**Supplementary Figure S5A**).

About 10.86% independent *cis*-eQTLs of lncRNAs was intercepted with known cancer risk loci (r^2^>0.5) (**Figure 3E**), which is much higher than that of *cis*-eQTLs of mRNAs (3.07%) reported recently (40), suggesting lncRNA is strongly related to GWAS cancer risk loci (**Supplementary Table S6** and **Figure S4C**). Herein, the *cis*-eQTLs of lncRNA were significantly enriched for GWAS cancer risk loci than for non-eQTLs (fold of enrichment =3.51, P<2.2 × 10^−16^) **(**For example, in ER-positive BRCA, we identified 12 *cis*-eQTLs which were in LD (r^2^>0.5) with breast cancer risk loci of rs62073257 (17q21.31), rs7104902 (11p15.5), and rs9393716 (6p22.2). In the case of *trans*-eQTLs, about 1.67% independent *trans*-eQTLs were intercepted with known cancer risk loci (**Figure 3E**) which corresponds to 7.74-fold of enrichment (P<2.2 × 10^−16^) compared with non-eQTLs. In the case of TAL, we observed that an average of 40.82% of *cis*-eQTLs of lncRNA were overlapped with TAL, while 27.89% of that of mRNA were intercepted with TAL (**Supplementary Table S6**).

### elncRNAs Enrich for lncRNAs with Known Clinical and Therapeutic Implications

**Figure 4:**
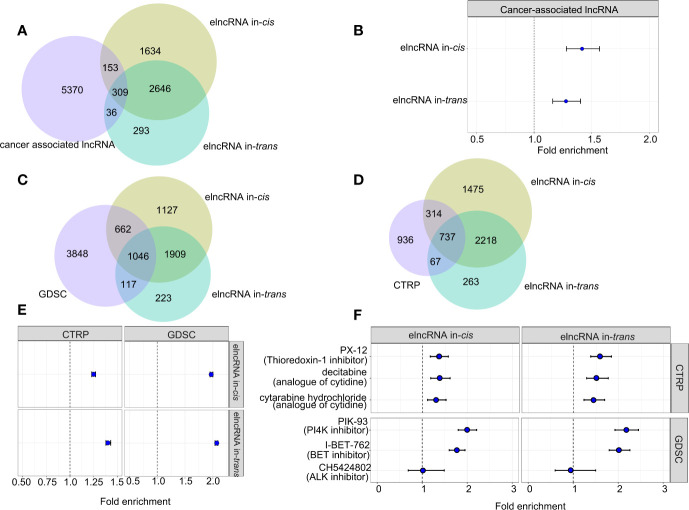
Association between elncRNAs, cancers and drugs. **(A)** Venn diagram showing the overlap among elncRNAs in-*cis*, elncRNAs in-*trans* and cancer-associated lncRNAs annotated by lncRNADisease v2.0. **(B)** Forrest plot showing the enrichment of elncRNAs in-*cis* and elncRNAs in-*trans* in cancer-associated lncRNAs. **(C)** Venn diagram showing the overlap among elncRNAs in-*cis*, elncRNAs in-*trans* and predictive lncRNAs for drugs in GDSC database. **(D)** Venn diagram showing the overlap among elncRNAs in-*cis*, elncRNAs in-*trans* and predictive lncRNAs for drugs in CTRP database. **(E)** Forrest plot showing the enrichment of elncRNAs in-*cis* and elncRNAs in-*trans* in predictive lncRNA for all drug sensitivity in two databases. **(F)** Forrest plot showing the enrichment of elncRNAs in-*cis* and elncRNAs in-*trans* in predictive lncRNA for the selective drug sensitivity in two databases (Fisher test).

To assess the clinical relevance of the elncRNAs, we retrieved the associations between the elncRNAs and the corresponding cancer types from the lncRNADisease v2.0 database (http://www.rnanut.net/lncrnadisease/). 462 of 4,742 (9.74%) elncRNAs in-*cis* and 679 of 3,284 (10.51%) elncRNAs in-*trans* were either proved or predicted to be associated with cancers from the lncRNADisease database (**Figure 4A**). Both elncRNA in-*cis* (OR=1.27, 95% CI: 1.16–1.40) and in-*trans* (OR=1.42, 95% CI:1.28–1.57) were significantly enriched in cancer-related lncRNAs (**Figure 4B**).

Recent studies suggest that lncRNA are associated with the treatment responses of cancers (21). Therefore, we evaluated the predictive power of the elncRNA in public databases contains drug IC50 for various cancer cell lines (21). As a result, both elncRNAs in-*cis* (OR=1.25, 95% CI: 1.23–1.27) and elncRNAs in-*trans* (OR=1.40, 95% CI: 1.38–1.43) were significantly enriched for lncRNAs which are proved predictive of the efficacy of anticancer drugs in the database of Cancer Therapeutics Response Portal (CTRP, https://portals.broadinstitute.org/ctrp/). Consistently, both elncRNAs in-*cis* (OR=1.97, 95% CI:1.95–2.00) and elncRNAs in-*trans* (OR=2.07, 95% CI:2.04 – 2.09) were enriched for the predictive lncRNAs in the database of Genomics of Drug Sensitivity in Cancer (GDSC, https://www.cancerrxgene.org/) (**Figures 4C, E**). In particular, we reported top drugs from each database with the highest significance of association to elncRNAs, namely, PX.12, decitabine, cytabarine hydrochloride in CTRP, and PIK-93 and I-BET-762 in GDSC (**Figures 4D, F**). There are also drugs which are specifically associated with elncRNAs in-*cis*, such as CH542802 (ALK inhibitor) in GDSC, and we found that except for CH542802 (ALK inhibitor), both the elncRNA in-*cis/trans* were significantly enriched in the predictive lncRNA sets of PX.12, decitabine, cytabarine hydrochloride in CTRP, and PIK-93 and I-BET-762 in GDSC (**Figures 4D, F**).

As prior studies show lncRNA strongly influence cancer immunity, we move on to evaluated the enrichment of elncRNAs in the annotated immunoreactive lncRNA database (23). As a result, both elncRNAs in-*cis/trans* were significantly overrepresented in the ImmLnc database (FDR<0.01). Moreover, the magnitude of enrichment of the *cis*-elncRNAs range from 1.20 fold (STAD) to 1.44 fold (THCA), while the enrichment for the *trans*-elncRNAs range from 1.18 fold (KIRC) to 1.47 fold (COAD) (**Supplementary Figure S5B**).

### The Downstream Transcripts of eQTL-elncRNA Influence Tumor Immune Microenvironment

We further investigated the impact of eQTLs-lncRNA associations on the downstream transcripts and the consequent pathological clinical phenotypes, such as the tumor immune microenvironment and the disease outcomes.

**Figure 5:**
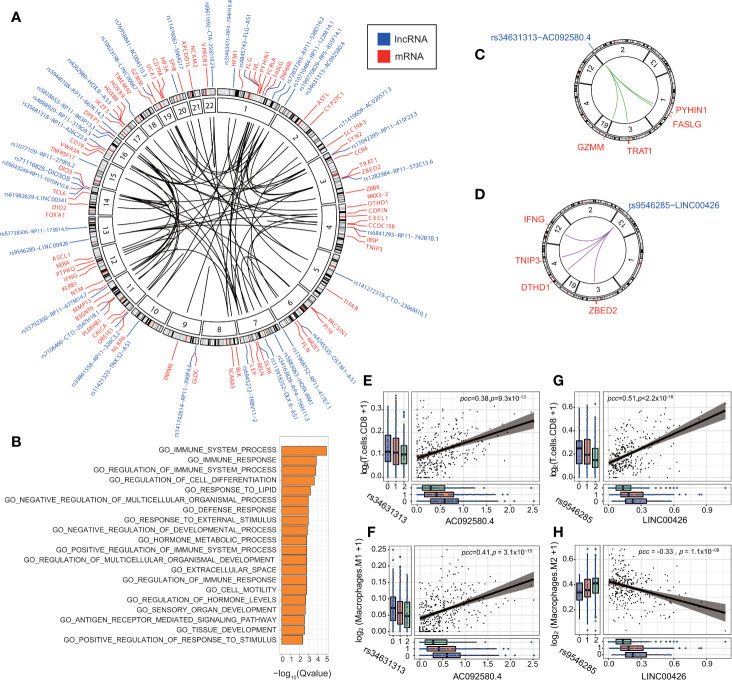
eQTL-elncRNA-mRNA regulatory axes are enriched in immune-related processes. **(A)** Circos plot showing the significant regulatory axes (inner circle) with FDR<0.1 and adjusted R^2^>0.1. the eQTL-elncRNA pairs are colored blue and the transcripts in-*trans* are colored in red. **(B)** Gene set enrichment analysis of the downstream target genes of the 191 eQTL-lncRNA-mRNA regulatory axes. **(C)** Illustration of the rs34631313(2p25.2)-AC092580.4-FASLG/GZMM/PYHIN1/TRAT1” regulatory axis in ovarian cancer. **(D)** Illustration of the rs9546285(13q12.3)-LINC00426-IFNG/TNIP3/DTHD1/ZBED2” regulatory axis in KIRC. **(E)** rs34631313(2p25.2)-AC092580.4 is positively associated with the fraction of tumor infiltrating CD8^+^ T cells in ovarian cancer. **(F)** rs34631313(2p25.2)-AC092580.4 is positively associated with the fraction of tumor infiltrating M1 macrophages in ovarian cancer. **(G)** rs9546285(13q12.3)-LINC00426 is positively associated with the fraction of tumor infiltrating CD8^+^ T cells in KIRC. **(H)** rs9546285(13q12.3)-LINC00426 is negatively associated with the fraction of tumor infiltrating M2 macrophages in KIRC.

We conducted IV analysis to identify the eQTL-elncRNA-mRNA regulatory axes in each cancer type. The IV regression takes the eQTL as an instrument to the independent variable (elncRNA in-*cis*), which in turn, affects the dependent variable (mRNA). Thus, we identified a total of 191 unique regulatory axes in 11 cancer types. **(**FDR<0.1; adjusted R^2^ >0.1, **Supplementary Table S7** and **Figure 5A**). We then evaluated the biological processes enriched in the downstream transcripts of the significant regulatory axes. As a result, we noticed that a set of the downstream target genes such as *IFNG, CALCA, CXCL1, NLRP6, BLK, CD79, FASLG, CCR4, GZMM*, were significantly enriched in the immune system process and immune response pathways (FDR<0.01 **Figure 5B**). This result prompted us to further study the impacts of eQTL-elncRNA on cancer immunity. We evaluated the effects of the 191 eQTL-elncRNA pairs on the fractions of tumor infiltrating immune cells in each TCGA cancer type estimated by prior studies (39). As a result, we identified 23 eQTL-elncRNA pairs in different cancer types (10 elncRNAs in 5 cancer types) which were significantly associated with the fraction of at least one immune cell subtype **(**P<0.05 and R^2^>0, **Supplementary Figure S7**). The eQTL-elncRNA pairs showed two opposite ways of impact (**Supplementary Figure S7**). In one way, AC092580.4 in OV and LINC00426 in KIRC were associated with lower fraction of Macrophage M2 and higher fraction of Macrophage M1 and CD8+ T cells (**Supplementary Figure S7**). M1 macrophages and CD8+ T cells are well known for their anti-tumoral effect, while M2 macrophages are reported as anti-inflammatory and associated with pro-tumor phenotypes (43). In the other way, RP4-756H11.3 in LIHC and RP11-677M14.7 in THCA were associated with increased fraction of M2 macrophages and decreased percentage of CD8+ T cells hence a pro-cancer effect (**Supplementary Figure S7**).

We further investigate the biological processes of the immune-related eQTL-elncRNA pairs. We showed that rs34631313 was a robust genetic instrument of AC092580.4 (P = 3.93 × 10^−4^), which influences a set of immune genes in-*trans*, including *FASLG* (effect size (β) = 1.53, P = 4.99 × 10^−9^), *GZMM* (β=1.20, P = 5.95 × 10^−6^), *PYHIN1* (β=1.54, P=3.42 × 10^−6^) and *TRAT1* (β = 1.42, P = 2.00 × 10^−5^). These genes are known to be involved in the regulation of immune system and cancer progression (44–47) (**Figure 5C**, **Supplementary Figure S6**). Consistently, the very same pair is also associated with higher fraction of CD8+ T cells (β= 0.095, P = 0.032) and M1 macrophages (β=0.065, P = 5.95 × 10^−6^) in ovarian cancer (**Figures 5E, F**).

In another case, rs9546285 (13q12.3) was a robust genetic instrument for LINC00426 and thereby influences an immune gene set of *TNIP3* (β=1.58, P=5.57 × 10^−5^), *ZBED2* (β=1.98, P=6.51 × 10^−8^), *IFNG* (β=1.58, P=1.78 × 10^−7^), and *DTHD1* (β=1.20, P=6.47 × 10^−6^) in-*trans* (**Figure 5D**, **Supplementary Figure S6**). These genes are involved in the antiviral and antitumor effects (48, 49). In KIRC, the same pair is associated with higher fraction of CD8+ T cells (β= 0.72, P = 6.34 × 10^−4^) and lower fraction of M2 macrophages (β = −0.39, P = 0.04) (**Figures 5G, H**). These results suggest that eQTLs of lncRNA influence the immune processes by regulating relevant genes and thus influence the constitutions of the tumor immune microenvironment.

### eQTL-elncRNA Regulatory Axis Associated With Cancer Clinical Outcome

**Figure 6:**
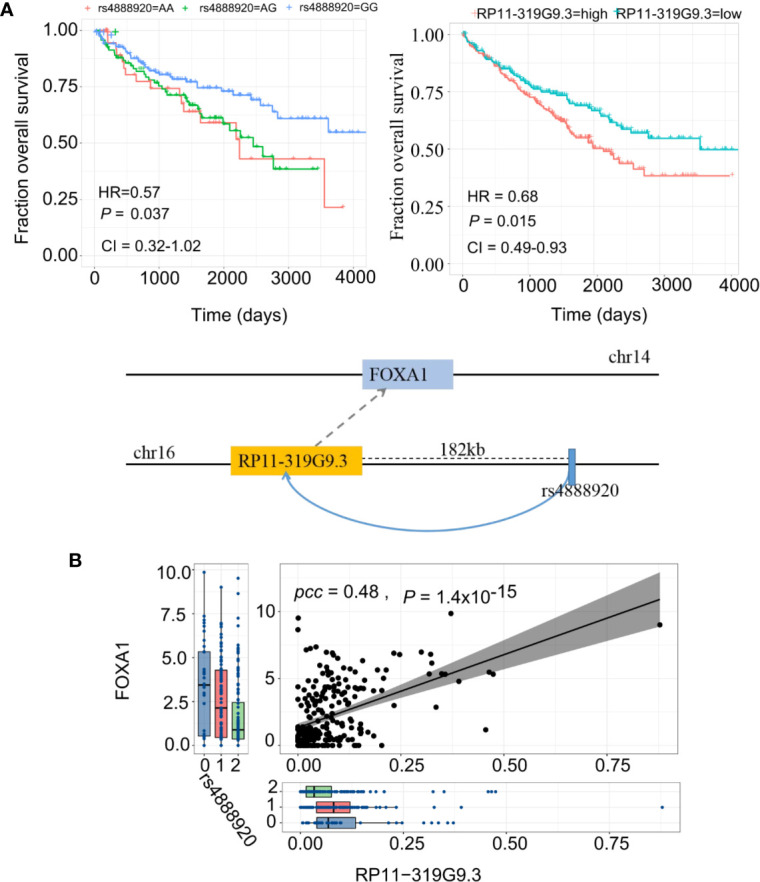
eQTL-elncRNA-mRNA regulatory axes predict clinical outcome of KIRC. **(A)** Illustration of the “rs4888920 (16q23.1)-RP11-319G9.3-FOXA1” regulatory axis in KIRC (lower panel) and the Kaplan-Meier curves based on overall survival of TCGA KIRC cohort stratified by the genotypes of rs4888920 (left) and the median expression levels of RP11-319G9.3 (right). **(B)** Correlations between rs4888920 (16q23.1)-RP11-319G9.3-FOXA1 in KIRC.

Finally, we report a regulatory axis, “rs4888920 (16q23.1)-RP11-319G9.3-FOXA1” (F-test for weak instrument, adjusted P=0.07, adjusted R^2^=0.14), with significant predictive power in KIRC **(****Figure 6A**). rs4888920 (16q23.1) was a *cis*-eQTL of RP11-319G9.3 (P=8.69 × 10^−6^) which is, in turn, positively associated with the expression of FOXA1 (β=2.06, P=6.12 × 10^−6^) (**Figure 6B**). Our data suggested that the eQTL (G/G vs. A/A and A/G: HR = 0.57, CI= [0.32, 1.02], P=0.037) and its elncRNA (RP11-319G9.3) in-*cis* (HR = 0.68, CI= [0.49, 0.93], P=0.015) are consistently significantly predictive of the overall survival in TCGA KIRC cohort (**Figure 6A**). In addition, we also found 23 eQTL-elncRNA pairs for FOXA1 in KIRC with significance and we listed the results in **Supplementary Table S8**.

## Discussion

The present study aimed to provide an in-depth view of the eQTL landscape of lncRNA in cancers by systematic assessment the downstream effects of the eQTL-elncRNA associations (40). Our data suggested that the genetic determinants of lncRNA expression are more active in cancer than those of mRNA, as the latter is directly associated with cancer phenotypes hence subject to higher selection pressure. Nevertheless, our results confirmed that the eQTL-elncRNA associations show more diverse but indirect effects on the phenotypes via the transcriptome such as the treatment responses (50) and immune microenvironments (51, 52).

While mapping the eQTLs in cancer, we accounted for different confounding effects (such as PEERs, SCNA, DNA methylations). Thus, our model is more stringent than those reported previously (11, 26), and the eQTLs directly interfered with the regulome of cancers. In addition, we demonstrated that the genomic landscape of *cis*-eQTLs of lncRNA was similar to that of the *cis*-eQTLs of mRNA. However, the *cis*-eQTL of lncRNA was greater than that of mRNAs in both *cis*-acting gene numbers per eQTL and effect sizes, which suggested that lncRNA expression is more susceptible to the genetic regulators than mRNA expression in cancers. Intriguingly, a number of cancer-related studies have demonstrated that germline risk variants effectuate via specific lncRNAs to impact tumor phenotypes (24, 53, 54). Such differences might be attributed to the different selection pressure in mammals (55) or different regulatory mechanisms underlying these two types of transcripts (56). Furthermore, the current data showed a significant higher enrichment of cancer risk loci in the eQTLs of lncRNAs than in those of mRNA in cancer, which is consistent with a previous study (26).

LncRNAs are known for a variety of regulatory activities in gene expression, many of which might exert functional and phenotypical impact in cancers. Therefore, we hypothesized that these elncRNAs can further interact with various downstream mRNA targets to influence the whole cancer transcriptome. Therefore, IV analysis was performed to identify regulatory axes in different cancer types. Especially, we report that the downstream transcripts of the elncRNAs in cancers were significantly enriched for the immunoregulatory processes. These results suggested that the eQTL act on immune genes *in trans* via the elncRNA. A previous study of *cis*-eQTL analysis of mRNA in cancers has also demonstrated that the *cis*-eQTL regulated mRNA are enriched in immunity pathways (40). We further conducted a second round of IV analysis, and 23 eQTLs-elncRNAs pairs were found to be significantly associated with at least one immune cell type. We closely analyzed the top representative eQTL-elncRNA pairs to determine the target immune genes and immune cell types of these pairs. These observations suggest germline variants have a strong impact on cancer immunity and may serve as predictive markers for immune therapy.

Notably, the top immune related eQTL-elncRNA pairs reported in the present study are also reported for association with drug sensitivity in prior independent studies (21). For example, LINC00426 is a strong predictor for Dexamethasone sensitivity in CCLE cell lines (21). Dexamethasone is a glucocorticoid (GC) steroid that is used as a supportive care co-medication for cancer patients undergoing standard pemetrexed/platinum doublet chemotherapy. It is used to reduce inflammation and suppress the body’s immune response by inhibition of *IL-2*, *IL-12*, and *IFN-γ* of signaling activities in NK, Th1, and CD8+ T cells (57). LINC000426 is associated with *IFNG* transcription and the activities of tumor infiltrating CD8+ T cells, hence LINC000426 expressing tumors are more sensitive to Dexamethasone. Since rs9546285 is a robust genetic regulator of LINC00426, this eQTL-elncRNA pair can inform the efficacy of Dexamethasone in cancer patients and also for cancer immunotherapy.

In addition, we identified several regulatory axes involving eQTL, elncRNA and mRNA by taking eQTLs as instrument variables. These regulatory axes cannot be easily identified by conventional *trans* association analysis due to the huge number of tests needed and the stringent control of false discovery rate. Many studies have shown that instrument variable regression, which is also known as Mendelian Randomization, is more efficient in identification of potential causal relationships between germline variants and different traits.

In certain cancer types, different eQTL-lncRNAs pairs influence the same set of mRNA targets; for example, in ovarian cancer, two eQTL (2p25.2 and 7q34) eventually regulate the same set of mRNAs (*FASLG, GZMM, PYHIN1 and TRAT1*) but through different elncRNAs (AC092580.4 and TRBV11-2). These results suggested that lncRNA act as a flexible intermediate between germline variants and gene expression in-*trans* and hence may play more active roles in cancers.

Nevertheless, the present study has several limitations. First, the inferred interaction patterns were based on multi-omics data, and further cellular and molecular experiments are needed to validate the findings. Then, the results were derived from 11 cancer types, and, thus, our findings may not apply to other cancer types. Third, the mapping of eQTLs of lncRNA was limited by the sample size, missing data, and confounding factors. The current results for the *trans*-eQTL hold limited statistical power due to a relatively small sample size (2549 samples). As a consequence, many eQTLs with small effect sizes cannot be identified. Also, we used the IV analyses to identify several regulatory axes with functional implications. However, these associations can occur in different cell types in the tumor tissue, or even via more complex intercellular interactions (58). Further functional analysis is needed to reveal the underlying biological mechanisms.

Taken together, the current findings provided insight into the genetic regulation of lncRNAs in 11 cancer types and explored the biological role as well as clinical phenotypes of eQTL-elncRNA in cancer immunology. Our findings may provide valuable genetic or lncRNA biomarkers for drug sensitivity and cancer immune therapy.

## Conclusions

This study investigated the eQTL landscape of lncRNAs in cancers and the potential biological function of elncRNAs in cancer microenvironment and drug sensitivity prediction. We performed instrumental analysis (Mendelian Randomization) to identify genes, pathways and immune cell types influenced by eQTL-elncRNA associations.

Our findings suggest that the downstream targets of eQTL-elncRNA pairs are enriched for immune system pathways and are consistently associated with varied fractions of immune cell types and patient clinical outcomes. Our data confirm that elncRNAs are active intermediates of non-coding genetic variants in cancer immunology and clinical outcome of cancers and provide valuable genetic and lncRNA biomarkers for drug sensitivity and cancer immune therapy.

## Data Availability Statement

Publicly available datasets were analyzed in this study. This data can be found here: The Cancer Genome Atlas (https://portal.gdc.cancer.gov/). The original contributions presented in the study are publicly available. This data can be found here: https://xcqxcq.github.io/lncRNA/LncRNA.html.

## Author Contributions

The project was conceived and directed by YZh and QL. Data analysis was performed by WL, CX, and YZh with assistance from JG, YZo, and ZWe. Image processing was carried out by CX, KL, and ZWa. The website for eQTL of lncRNA was constructed by CX, YH, HF, and DW. The manuscript was written by YZh and QL. All authors contributed to the article and approved the submitted version.

## Funding

This work was supported by the National Natural Science Foundation of China [81802823 to YZh]; the Fundamental Research Funds for the Chinese Central Universities [20720190101 to QL]; the Natural Science Foundation of Fujian Province of China [2018J01054 to YZh]; the Major Project of Shanghai Science and Technology Commission of China [18441901700 to WL]; and the Joint Project of Major and Critical Diseases Funded by Xiamen Municipal Health and Family Planning [3502Z20179053 to DW].

## Conflict of Interest

The authors declare that the research was conducted in the absence of any commercial or financial relationships that could be construed as a potential conflict of interest.
