# Supplementary material for: Cis- and Trans-Acting Expression Quantitative Trait Loci of Long Non-Coding RNA in 2,549 Cancers With Potential Clinical and Therapeutic Implications
Source: Front Oncol. 2020 Oct 19;10:602104. doi: 10.3389/fonc.2020.602104 (PMC7604522; doi:10.3389/fonc.2020.602104)
Supplement: Supplementary file 1 [file DataSheet_1.pdf]

Figures S1-S7

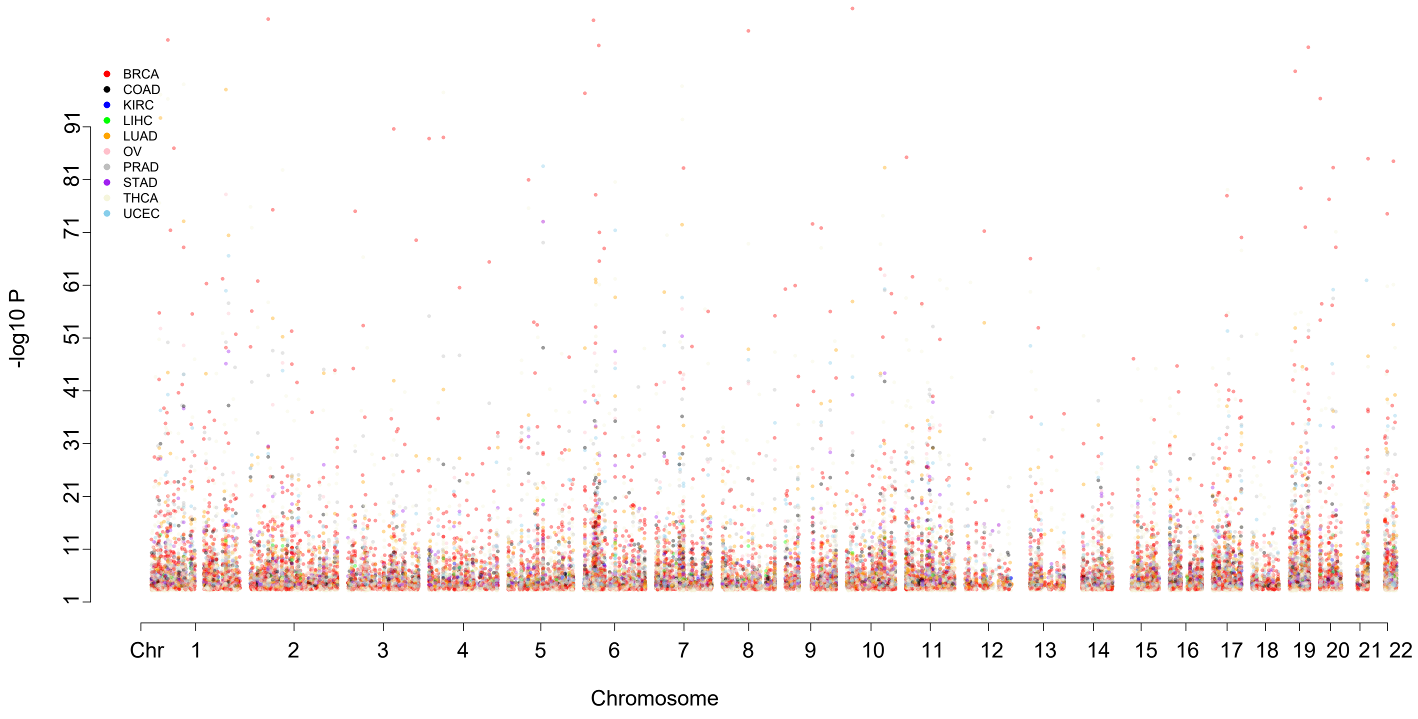

Figure S1 Manhattan plot of *cis*-eQTLs of mRNA in 10 cancer types

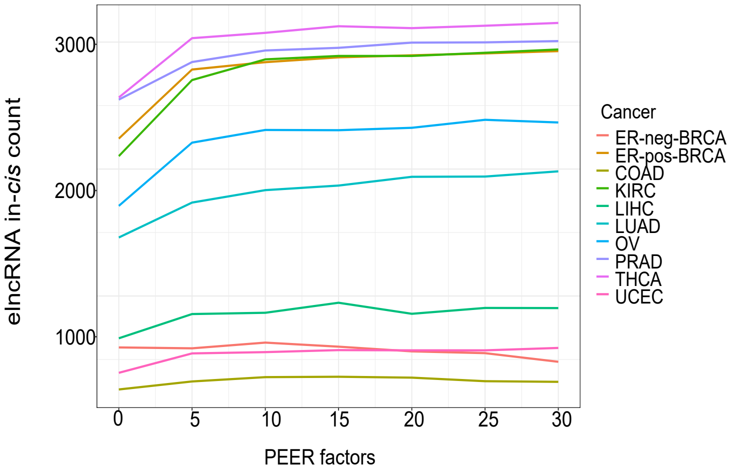

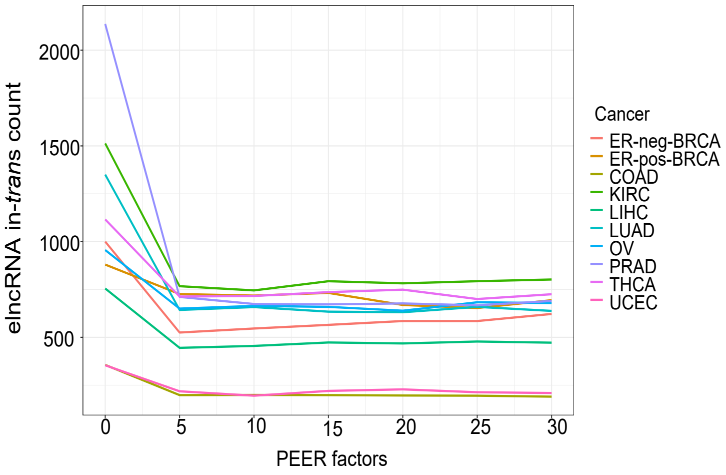

Figure S2 elncRNA in *cis/trans* count using different PEER factors

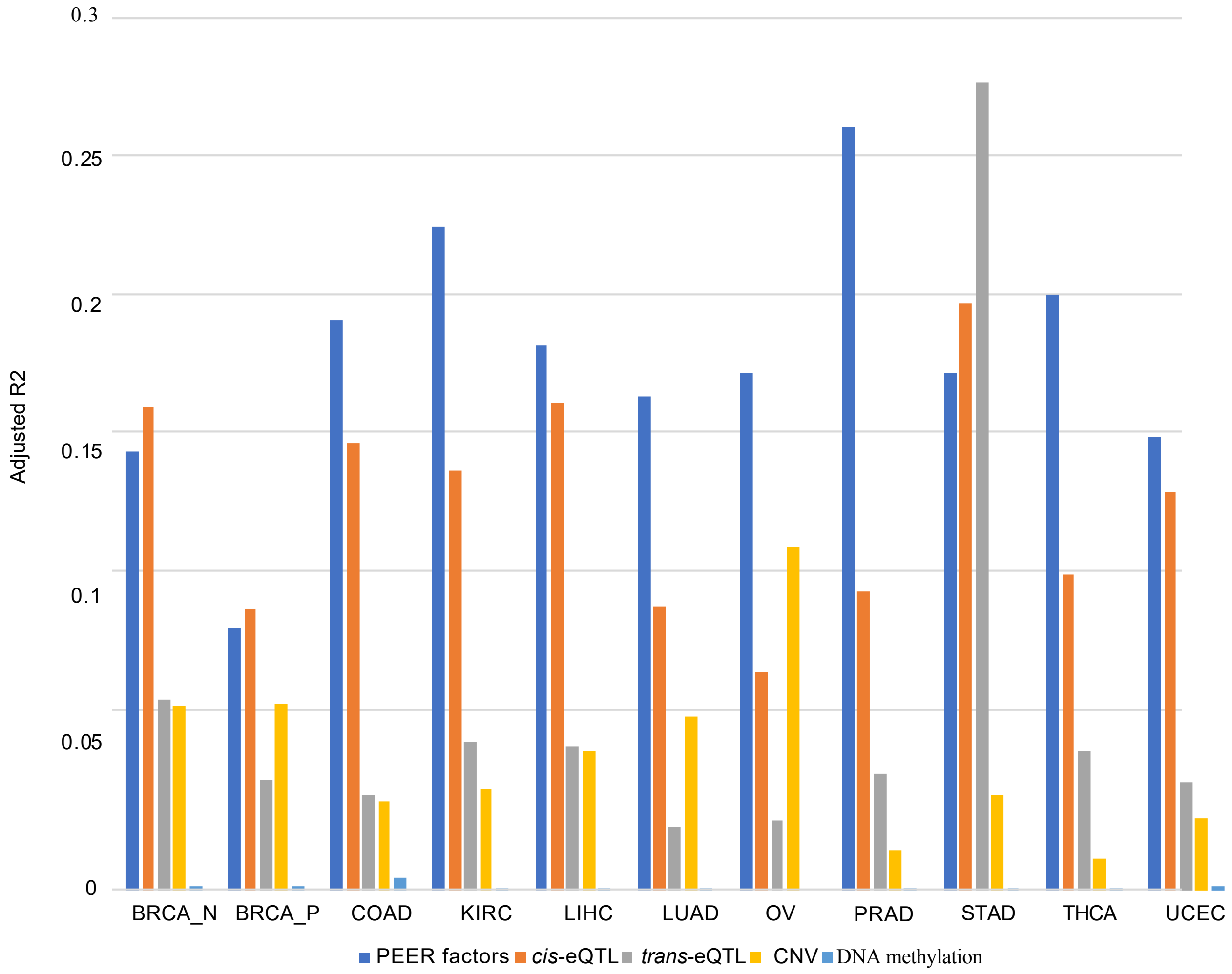

Figure S3 Comparison of the fractions of variation of lncRNA expression explained by the major factors

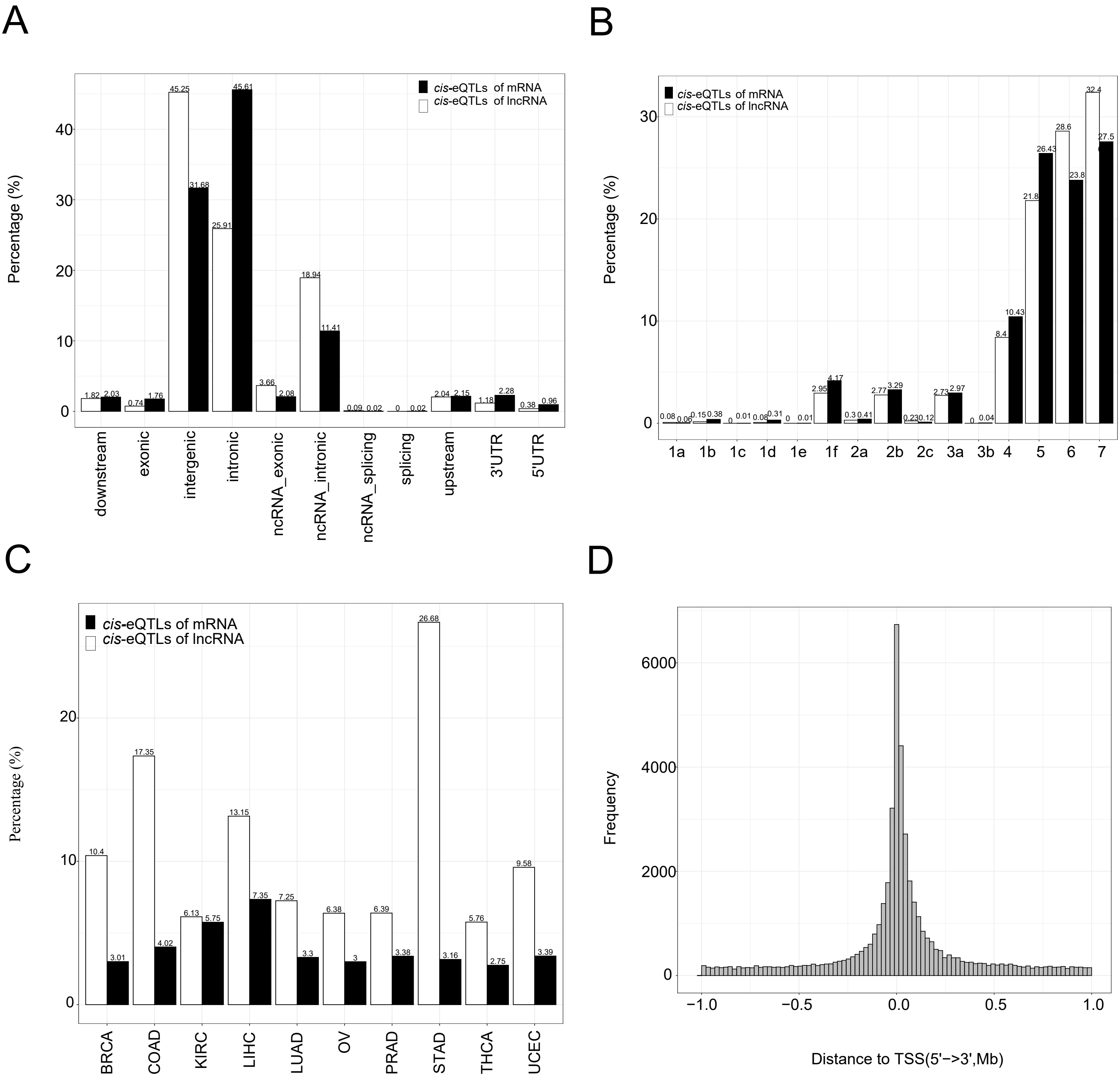

Figure S4 Characterization and comparison of *cis*-eQTLs of mRNA and lncRNA

(A) Functional effects of cis-eQTLs on mRNA/lncRNA (r2≥ 0.2).
(B) Distribution of RegulomeDB categories of cis-eQTLs of mRNA/lncRNA (r2≥ 0.2).
(C) Distribution of cis-eQTLs of mRNA/lncRNA associated with GWAS cancer-related risk loci.
(D) Location distribution of significant cis-eQTLs relative to the mRNAs aggregated across all cancer types.

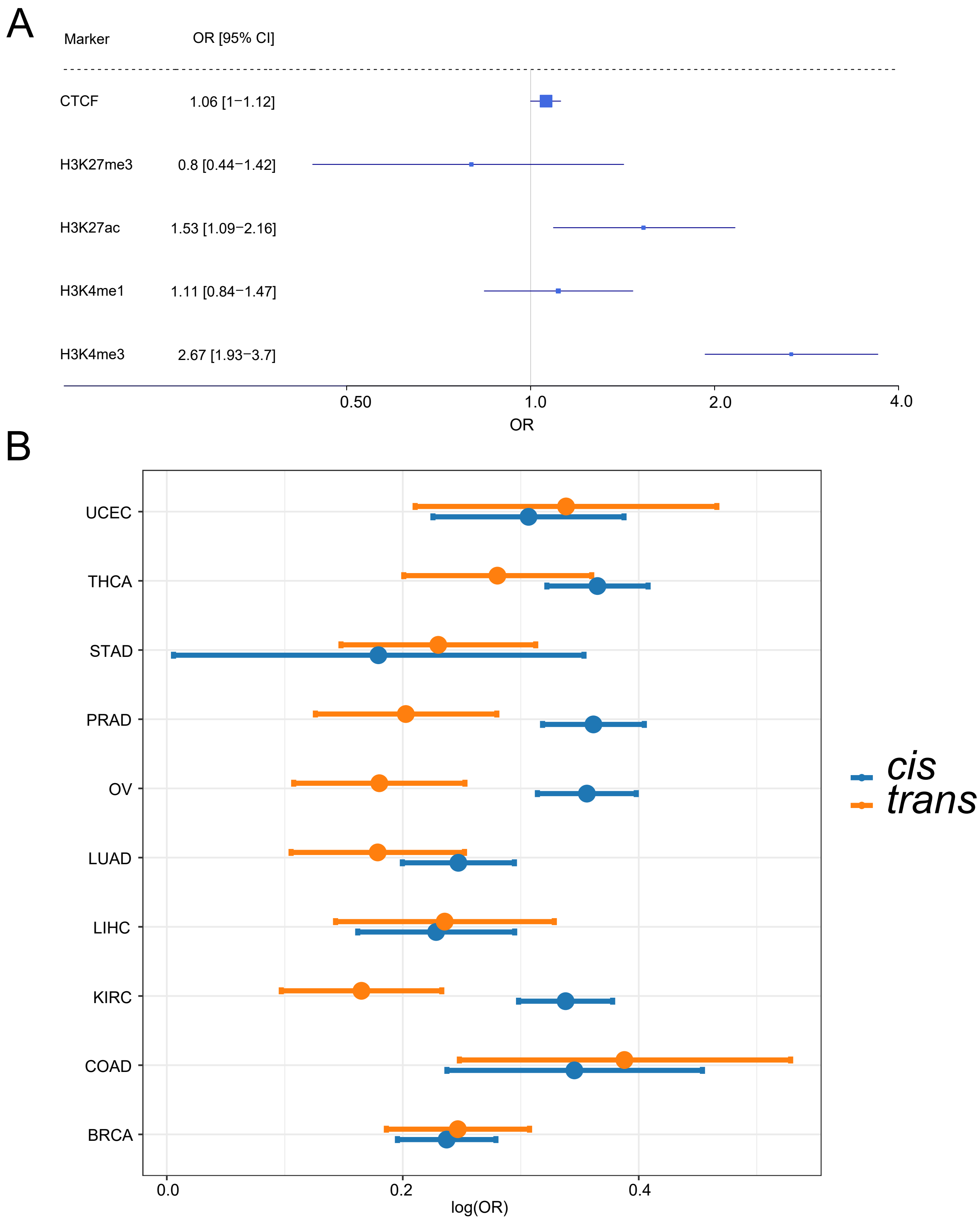

Figure S5

(A) elncRNAs that are significantly overlapped for immune-related lncRNAs

(B) meta-analysis of corresponding normal tissues showing the enrichment of *cis*-eQTLs in different epigenetic markers.

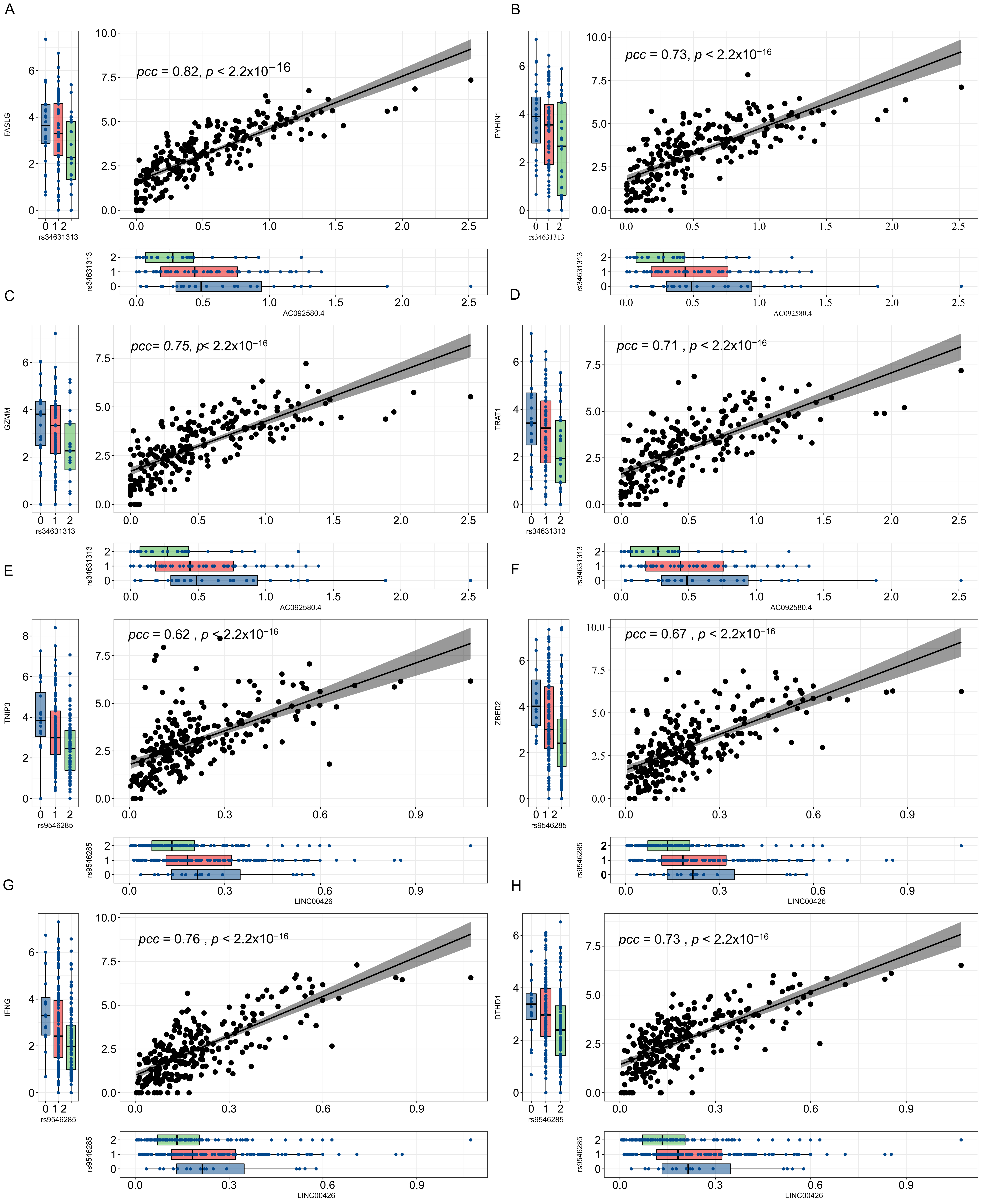

Figure S6 (**A-D)** Correlations among rs34631313(2p25.2)-AC092580.4-FASLG/GZMM/PYHIN1/TRAT1 regulatory axis in ovarian cancer (OV). (**E-H)** Correlations among rs9546285(13q12.3)-LINC00426-TNIP3/ZBED2/PYHIN1/DTHD1 regulatory axis in Kidney Renal Clear Cell Carcinoma (KIRC).

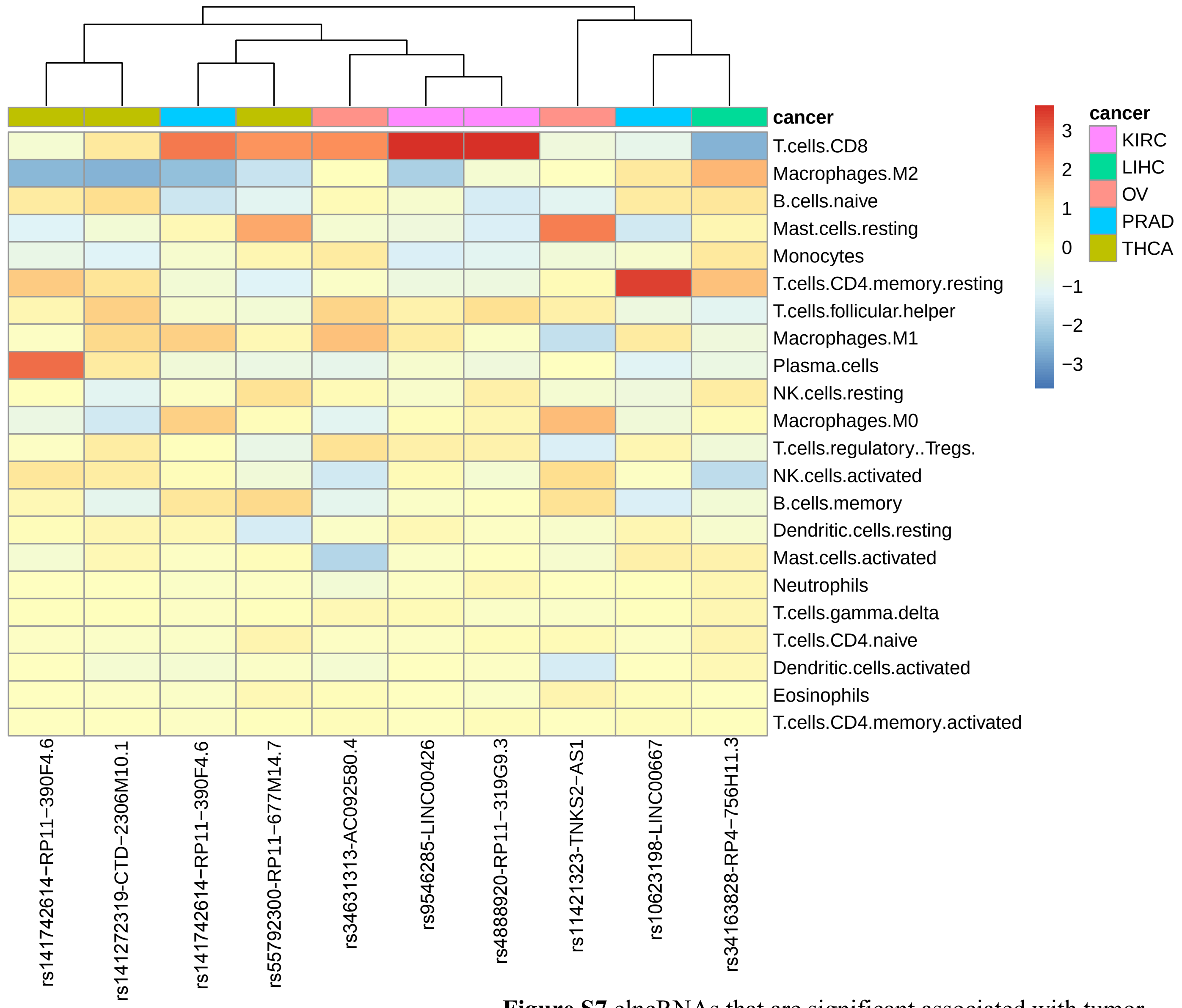

Figure S7 elncRNAs that are significant associated with tumor infiltrating immune cell fractions from IV analysis ($P<0.05$, $R^2>0$)
