# Supplementary material for: Cis- and Trans-Acting Expression Quantitative Trait Loci of Long Non-Coding RNA in 2,549 Cancers With Potential Clinical and Therapeutic Implications
Source: Front Oncol. 2020 Oct 19;10:602104. doi: 10.3389/fonc.2020.602104 (PMC7604522; doi:10.3389/fonc.2020.602104)
Supplement: Supplementary file 2 [file Table_1.docx]

| **Table S1.** Description of regulomeDB score | |
| --- | --- |
| Score | Supporting data |
| 1a | eQTL + TF binding + matched TF motif + matched DNase Footprint + DNase peak |
| 1b | eQTL + TF binding + any motif + DNase Footprint + DNase peak |
| 1c | eQTL + TF binding + matched TF motif + DNase peak |
| 1d | eQTL + TF binding + any motif + DNase peak |
| 1e | eQTL + TF binding + matched TF motif |
| 1f | eQTL + TF binding / DNase peak |
| 2a | TF binding + matched TF motif + matched DNase Footprint + DNase peak |
| 2b | TF binding + any motif + DNase Footprint + DNase peak |
| 2c | TF binding + matched TF motif + DNase peak |
| 3a | TF binding + any motif + DNase peak |
| 3b | TF binding + matched TF motif |
| 4 | TF binding + DNase peak |
| 5 | TF binding or DNase peak |
| 6 | other |
| 7 | not available |
