# Supplementary material for: Cis- and Trans-Acting Expression Quantitative Trait Loci of Long Non-Coding RNA in 2,549 Cancers With Potential Clinical and Therapeutic Implications
Source: Front Oncol. 2020 Oct 19;10:602104. doi: 10.3389/fonc.2020.602104 (PMC7604522; doi:10.3389/fonc.2020.602104)
Supplement: Supplementary file 3 [file Table_2.docx]

| **Table S2.** Cancer type and sample size summary | | | |
| --- | --- | --- | --- |
| Cancer Type | Expr+age+gender+peers+SNP | Expr+age+gender+peers+SNP+CNV | Expr+age+gender+peers+SNP+CNV+Methy |
| ER-negative-BRCA | 96 | 96 | 59 |
| ER-positive-BRCA | 459 | 459 | 282 |
| COAD | 146 | 146 | 26 |
| KIRC | 251 | 251 | 155 |
| LIHC | 113 | 113 | 113 |
| LUAD | 249 | 249 | 216 |
| OV | 331 | 331 | 0 |
| PRAD | 283 | 283 | 283 |
| STAD | 42 | 42 | 42 |
| THCA | 345 | 345 | 345 |
| UCEC | 234 | 234 | 160 |
