# Supplementary material for: Cis- and Trans-Acting Expression Quantitative Trait Loci of Long Non-Coding RNA in 2,549 Cancers With Potential Clinical and Therapeutic Implications
Source: Front Oncol. 2020 Oct 19;10:602104. doi: 10.3389/fonc.2020.602104 (PMC7604522; doi:10.3389/fonc.2020.602104)
Supplement: Supplementary file 4 [file Table_3.docx]

| **Table S3.** Statistics of *cis*-eQTLs of lncRNA and *cis*-eQTLs of mRNA across cancer types | | | | | | | | | |
| --- | --- | --- | --- | --- | --- | --- | --- | --- | --- |
| cancer | *cis*-eQTLs of lncRNA | *cis*-elncRNA | *cis*-eQTLs of lncRNA/*cis*-elncRNA | *cis*-eQTLs of mRNA | *cis*-emRNA | *cis*-eQTLs of mRNA/*cis*-emRNA | number of overlapped eQTLs | | percentage of overlapped eQTLs |
| BRCA | 21188 | 2805 | 7.55 | 11410 | 8413 | 1.36 | 152 | 0.007 | |
| COAD | 1458 | 313 | 4.66 | 3056 | 2490 | 1.23 | 7 | 0.005 | |
| KIRC | 15486 | 2613 | 5.93 | 226 | 226 | 1 | 1 | 6.46e-05 | |
| LIHC | 3877 | 817 | 4.75 | 313 | 295 | 1.06 | 7 | 0.00181 | |
| LUAD | 8761 | 1672 | 5.24 | 5605 | 4232 | 1.32 | 46 | 0.0053 | |
| OV | 11976 | 2125 | 5.64 | 3403 | 2638 | 1.29 | 58 | 0.0048 | |
| PRAD | 21547 | 2779 | 7.76 | 7066 | 5594 | 1.26 | 97 | 0.0045 | |
| STAD | 461 | 98 | 4.70 | 2440 | 1918 | 1.27 | 1 | 0.002 | |
| THCA | 28085 | 2970 | 9.46 | 13344 | 10087 | 1.32 | 180 | 0.0064 | |
| UCEC | 2976 | 527 | 5.65 | 3949 | 3023 | 1.31 | 17 | 0.0057 | |
