# Supplementary material for: Cis- and Trans-Acting Expression Quantitative Trait Loci of Long Non-Coding RNA in 2,549 Cancers With Potential Clinical and Therapeutic Implications
Source: Front Oncol. 2020 Oct 19;10:602104. doi: 10.3389/fonc.2020.602104 (PMC7604522; doi:10.3389/fonc.2020.602104)
Supplement: Supplementary file 5 [file Table_4.docx]

| **Table S4.** Summary of elncRNAs associated with both *cis*- and *trans*-eQTLs | | | |
| --- | --- | --- | --- |
| Cancer type | elncRNA in-*cis* which overlap with elncRNA in-*trans* (count) | total elncRNA count | percentage |
| ER-neg-BRCA | 77 | 966 | 7.97 |
| ER-pos-BRCA | 517 | 2873 | 18 |
| COAD | 54 | 449 | 12.03 |
| KIRC | 487 | 2851 | 17.08 |
| LIHC | 133 | 1107 | 12.01 |
| LUAD | 290 | 1987 | 14.59 |
| OV | 346 | 2386 | 14.5 |
| PRAD | 510 | 2940 | 17.35 |
| STAD | 18 | 531 | 3.39 |
| THCA | 569 | 3069 | 18.54 |
| UCEC | 106 | 624 | 16.99 |
| total | 3107 |  |  |
