# Supplementary material for: Cis- and Trans-Acting Expression Quantitative Trait Loci of Long Non-Coding RNA in 2,549 Cancers With Potential Clinical and Therapeutic Implications
Source: Front Oncol. 2020 Oct 19;10:602104. doi: 10.3389/fonc.2020.602104 (PMC7604522; doi:10.3389/fonc.2020.602104)
Supplement: Supplementary file 6 [file Table_5.docx]

| **Table S5.** Summary of eQTLs that act both in-*cis* and in-*trans* | | | | | |
| --- | --- | --- | --- | --- | --- |
| Cancer type | Overlap count | *Cis*-eQTL count | *Trans*-eQTL count | *Cis*-percentage | *Trans*-percentage |
| ER-neg-BRCA | 1 | 2765 | 754 | 0.04 | 0.13 |
| ER-pos-BRCA | 218 | 20379 | 1844 | 1.07 | 11.82 |
| COAD | 2 | 1458 | 325 | 0.14 | 0.62 |
| KIRC | 73 | 15486 | 1465 | 0.47 | 4.98 |
| LIHC | 2 | 3877 | 634 | 0.05 | 0.32 |
| LUAD | 3 | 8761 | 1114 | 0.03 | 0.27 |
| OV | 226 | 11976 | 1292 | 1.89 | 17.49 |
| PRAD | 199 | 21547 | 1606 | 0.92 | 12.39 |
| STAD | 0 | 461 | 617 | 0 | 0 |
| THCA | 315 | 28085 | 2183 | 1.12 | 14.43 |
| UCEC | 0 | 2976 | 292 | 0 | 0 |
| total | 1039 |  |  |  |  |
