# Supplementary material for: Cis- and Trans-Acting Expression Quantitative Trait Loci of Long Non-Coding RNA in 2,549 Cancers With Potential Clinical and Therapeutic Implications
Source: Front Oncol. 2020 Oct 19;10:602104. doi: 10.3389/fonc.2020.602104 (PMC7604522; doi:10.3389/fonc.2020.602104)
Supplement: Supplementary file 7 [file Table_6.docx]

| **Table S6.** *cis*-eQTLs comparison between lncRNA and mRNA | | | |
| --- | --- | --- | --- |
|  | *cis*-eQTL (lncRNA) | *cis*-eQTL (mRNA) | overlap |
| rho (the number of significant *cis*-eQTLs v.s. sample size) | 0.87 | 0.94 |  |
| average effect size of *cis*-eQTLs | 0.54 | 0.37 |  |
| cancer risk loci-related eQTL percentage | 0.1086 | 0.0307 |  |
| trait associated loci eQTL percentage | 0.4082 | 0.2789 |  |
| average fraction of tumor gene expression variance explained by *cis*-eQTLs | 0.1093 | 0.053 |  |
| the number of eQTL | 59542 | 64094 | 1571 |
| the number of elncRNA/emRNA | 4742 | 18210 | 587 |
