# Supplementary material for: Cis- and Trans-Acting Expression Quantitative Trait Loci of Long Non-Coding RNA in 2,549 Cancers With Potential Clinical and Therapeutic Implications
Source: Front Oncol. 2020 Oct 19;10:602104. doi: 10.3389/fonc.2020.602104 (PMC7604522; doi:10.3389/fonc.2020.602104)
Supplement: Supplementary file 9 [file Table_8.docx]

| **Table S8.** All 23 significant eQTL-elncRNA pairs for FOXA1 were reported in KIRC | | | | | |
| --- | --- | --- | --- | --- | --- |
| Cancer | SNP | Hgnc symbol | Mrna | LncRNA_P | Iv_p |
| KIRC | rs72308607 | SNHG17 | FOXA1 | 0.0386 | 0.000016 |
| KIRC | rs35070476 | LINC00571 | FOXA1 | 0.0232 | 0.0000201 |
| KIRC | rs373888828 | LINC00571 | FOXA1 | 0.0464 | 0.0000537 |
| KIRC | rs5802927 | LINC00571 | FOXA1 | 0.0464 | 0.0000537 |
| KIRC | rs5802928 | LINC00571 | FOXA1 | 0.0374 | 0.0000116 |
| KIRC | rs148869466 | LINC00571 | FOXA1 | 0.0419 | 0.000377 |
| KIRC | rs150564754 | LINC00571 | FOXA1 | 0.0464 | 0.0000537 |
| KIRC | rs199546061 | LINC00571 | FOXA1 | 0.0244 | 0.000104 |
| KIRC | rs35062568 | LINC00571 | FOXA1 | 0.0464 | 0.0000537 |
| KIRC | rs10611968 | LINC00571 | FOXA1 | 0.0464 | 0.0000537 |
| KIRC | rs10646965 | LINC00571 | FOXA1 | 0.0464 | 0.0000537 |
| KIRC | rs145682229 | LINC00571 | FOXA1 | 0.0413 | 0.000386 |
| KIRC | rs11435100 | LINC00571 | FOXA1 | 0.0419 | 0.000377 |
| KIRC | rs117818215 | LINC00571 | FOXA1 | 0.0413 | 0.000386 |
| KIRC | rs145298261 | LINC00571 | FOXA1 | 0.0413 | 0.000386 |
| KIRC | rs11453215 | LINC00571 | FOXA1 | 0.0212 | 0.0000775 |
| KIRC | rs79993226 | LINC00337 | FOXA1 | 0.0462 | 0.0000283 |
| KIRC | rs76200612 | RP11-557H15.4 | FOXA1 | 0.0331 | 0.000169 |
| KIRC | rs11054078 | RP11-434C1.1 | FOXA1 | 0.0366 | 6.45E-26 |
| KIRC | rs11388301 | AC226118.1 | FOXA1 | 0.0304 | 0.000017 |
| KIRC | rs147847566 | RP11-158M2.4 | FOXA1 | 0.037 | 0.00218 |
| KIRC | rs34859851 | RP11-114H24.6 | FOXA1 | 0.0479 | 0.0000158 |
| KIRC | rs74472684 | AC002044.4 | FOXA1 | 0.0479 | 0.000404 |
